# Correction to ‘Expression of mRNA electroporated into plant and animal cells’

**DOI:** 10.1093/nar/gkab636

**Published:** 2021-07-21

**Authors:** J Callis, M Fromm, V Walbot

**Affiliations:** Department of Biological Sciences, Stanford University, Stanford, CA 94305, USA; Department of Biological Sciences, Stanford University, Stanford, CA 94305, USA; Department of Biological Sciences, Stanford University, Stanford, CA 94305, USA

In the original HTML version of this article (1), there was an error in the spelling of first author J. Callis's surname. The full name should read: ‘J. Callis’ instead of ‘J. Cellis’. This error does not appear in the PDF version of the article and has been corrected only in the HTML version with this erratum to preserve the published version of record.

## Notes

Present address: J. Callis, Horticulture Department, University of Wisconsin, Madison, WI 53706, USA.

Present address: M. Fromm, USDA-PGEC, 800 Buchanan Street, Albany, CA 94710, USA.
